# Potential impacts of climate change on habitat suitability for the Queensland fruit fly

**DOI:** 10.1038/s41598-017-13307-1

**Published:** 2017-10-12

**Authors:** Sabira Sultana, John B. Baumgartner, Bernard C. Dominiak, Jane E. Royer, Linda J. Beaumont

**Affiliations:** 1 Department of Biological Sciences, Macquarie University, North Ryde, New South Wales 2109 Australia; 2 New South Wales Department of Primary Industries, Locked Bag 21, Orange, New South Wales 2800 Australia; 3 Queensland Department of Agriculture and Fisheries, Biosecurity Queensland, Brisbane, Queensland 4001 Australia

## Abstract

Anthropogenic climate change is a major factor driving shifts in the distributions of pests and invasive species. The Queensland fruit fly, *Bactrocera tryoni* (Froggatt) (Qfly), is the most economically damaging insect pest of Australia’s horticultural industry, and its management is a key priority for plant protection and biosecurity. Identifying the extent to which climate change may alter the distribution of suitable habitat for Qfly is important for the development and continuation of effective monitoring programs, phytosanitary measures, and management strategies. We used Maxent, a species distribution model, to map suitable habitat for Qfly under current climate, and six climate scenarios for 2030, 2050 and 2070. Our results highlight that south-western Australia, northern regions of the Northern Territory, eastern Queensland, and much of south-eastern Australia are currently suitable for Qfly. This includes southern Victoria and eastern Tasmania, which are currently free of breeding populations. There is substantial agreement across future climate scenarios that most areas currently suitable will remain so until at least 2070. Our projections provide an initial estimate of the potential exposure of Australia’s horticultural industry to Qfly as climate changes, highlighting the need for long-term vigilance across southern Australia to prevent further range expansion of this species.

## Introduction

The Queensland fruit fly (Qfly), *Bactrocera tryoni* (Froggatt), is the most devastating pest of Australia’s $9 billion p.a. horticulture industry. Endemic to north-eastern Australia, its range expanded southwards following the planting of exotic horticultural crops^1^. Populations now span eastern Australia from the Cape York Peninsula in far north-east Queensland, through New South Wales (NSW) and into the southern state of Victoria where its range has been reported to be restricted by low precipitation and temperature to the west and south, respectively^2^. Qfly has also achieved serious pest status in the north of the Northern Territory^3^, although it is unclear whether these populations consist of Qfly or a fertile hybrid with *Bactrocera aquilonis*^4^. In the west, the climate of Perth and surrounds are suitable for Qfly^5^ with outbreaks occurring during 1989–1990^6^. Although this resulted in an extensive and successful eradication campaign, several incursions have occurred since^7^. Within urban South Australia, Qfly outbreaks have occurred due to the entry of infested fruits from other states^8^. Currently, Tasmania is the only state where Qfly outbreaks do not occur^9^. As such, it is recognised for ‘Area-Freedom’ from fruit flies. Consequently, crop production costs are lower as produce does not require costly disinfestation procedures before being exported^10^, and this adds considerably to the value of the state’s horticultural industry^9^.

Qfly attacks more than 100 native and introduced host plant species^11,12^, including citrus, pome and stone fruits, berries and tropical fruits, and ‘fruiting vegetables’. The economic costs of this pest are considerable. Abdalla *et al*.^13^ estimated the annual cost of pre-harvest bait and cover spraying over the period 2006–2009 to be ~$48 million, while post-harvest treatments (which may include chemical fumigants, temperature treatments, or irradiation)^14^ necessary to transport produce interstate exceeded $22 million p.a. Even with these treatments, production losses in fruit fly endemic regions range from 0.5–3%^13^. The above figures do not include costs to backyard growers (which in the absence of eradication programs could result in 80% of the value of backyard fruit production being lost^15^), costs of restricted access to domestic or international markets, and flow-on costs to related industries, such as food retailers and processors, or the wine industry^13^.

Given the costs of Qfly and other fruit flies to the horticultural industry, the Tri-state Fruit Fly Exclusion Zone (FFEZ) was established in 1994, spanning the major fruit growing regions of south-western New South Wales, north-western Victoria and south-eastern South Australia^16^. In an endeavour to keep the FFEZ free of fruit flies, and thereby maintain high value markets, there were stringent legislative controls on the transport of fruit and vegetables into this region. However, in 2010–2011, the FFEZ was subjected to the wettest two-year period on record, and outbreaks occurred in the NSW and Victorian parts of the FFEZ. Control and eradication measures became technically unfeasible and economically unsustainable. By August 2013, the legislation supporting the FFEZ was withdrawn in NSW and Victoria, and the Zone ceased to be a trade zone (Dominiak and Mapson, accepted). The Sunraysia Pest Free Area stills exists in the northwest corner of the FFEZ, although this zone is currently suspended.

As with other insects, the distribution, abundance and development rate of Qfly are strongly influenced by climate. In particular, there is a strong positive correlation between summer rainfall and Qfly abundance^4,17,18^, with O’Loughlin^19^ noting that abundance increases significantly when summer rainfall exceeds 170 mm per month. Without rainfall, the fecundity of adult females declines, mortality of larvae and newly emerged adults increases, and there may be markedly diminished emigration to nearby regions^18^. Temperature also influences the distribution and development of Qfly^18^. The critical lower temperature, below which individuals cannot move spontaneously, is ~2 °C, and although adults may survive at temperatures of 38–40 °C ^2,17^, immature stages are more vulnerable to such extremes^20^.

Given the dependence of Qfly distribution and abundance on climate variables, there is concern that as climate change intensifies, warmer temperatures and changes to precipitation patterns will facilitate the spread of populations southward and into Tasmania^21^. There is also the potential for more frequent outbreaks to occur within the former FFEZ and in other Australian horticultural regions.

Previous studies using the mechanistic species distribution model, CLIMEX, have estimated the potential for Qfly to undergo increases in population sizes and range expansion as a result of climate change^4,9,10^. In particular, warmer winters may increase the survival and development rates of Qfly, resulting in greater population numbers in spring^10^. While highly useful in furthering our understanding of climate impacts on Qfly, these publications were either restricted in geographic scope (e.g. to Tasmania^9^) or are now somewhat dated, as the development of climate models and greenhouse gas concentration pathways has advanced considerably since their publication, as has the availability of data, the sophistication of modelling tools, and spatial resolution of analyses. As such, here we employ the species distribution model (SDM) Maxent to conduct a continent-wide assessment of the potential impacts of climate change on Qfly. Maxent is a correlative SDM that has been used extensively to assess the distribution of suitable habitat for a broad range of pest and invasive species^22,23^. Our goal is to assess how climate change may impact the distribution of suitable habitat for Qfly, across a range of plausible climate scenarios. Furthermore, we assess the extent to which the FFEZ and other Australian horticultural areas may be suitable for Qfly in 2030, 2050 and 2070. Our study provides essential foundations for a broad understanding of the potential exposure of Australia’s horticultural industry to Qfly incursions in the future.

## Methodology

### Species data

We obtained occurrence data for Qfly from four main sources: the Atlas of Living Australia (ALA; http://www.ala.org.au), the Australian Plant Pest Database (http://www.planthealthaustralia.com.au/resources/australian-plant-pest-database), existing literature, and trap data. ALA is Australia’s largest digital database of species occurrence records, containing information from a wide array of data providers including Australia’s major museums and government departments. Before downloading data from ALA, we applied filters to restrict records to those that were resolved to species-level, dated after 1 January 1950, contained geographic coordinates, and were not flagged as ‘environmental outliers’. APPD is a national, secure database of pest and plant pathogen specimens held within herbaria and insect collections across Australia. Records from ALA and APPD primarily represent ad hoc collections, and so were supplemented with records from specimens collected in fruit fly traps managed by various state government departments (New South Wales Regional Pest Management, Biosecurity and Food Safety; Biosecurity Queensland and the Queensland Department of Agriculture and Fisheries; Department of Economic Development, Jobs, Transport and Resources, Victoria; and Primary Industries and Regions South Australia (PIRSA)). Trap data from these sources were collected at different periods from 1996 to 2017. To reduce environmental bias due to spatially autocorrelated sampling, we reduced trap data such that pairs of points were separated by at least 10 km. We also obtained occurrence data from previous studies^24–27^ including state government databases. After filtering/thinning, a total of 1057 unique localities (i.e. 1 × 1 km grid cells) remained.

### Current habitat data

For current climatic conditions (1950–2000) we downloaded 19 ‘bioclimatic’ variables from the WorldClim database^28^ (http://www.worldclim.org/) at a spatial resolution of 30 arc-seconds. We assessed pairwise correlations among these variables, and generated three sets of variables with Pearson correlation coefficients having absolute values < 0.8. We supplemented the climate variables with data on soil characteristics, available from the CSIRO data access portal (https://data.csiro.au). These variables were developed by Viscarra Rossel & Chen^29^ from a principal components analysis of visible and near infrared soil spectra, and are referred to as PC1, PC2 and PC3. They describe, respectively, the distribution of highly weathered soils, soils with large amounts of organic matter, and low relief landscapes with soils containing abundant smectite (clay) minerals^29^.

Finally, we developed multiple Maxent models based on different combinations of the climate and soil variables, to identify the subset that resulted in models with the highest predictive power (AUC, described below). Ultimately, we selected the following variables for our final model: mean annual temperature (MAT), minimum temperature of the coldest month (TminCM), temperature annual range (TAR), precipitation of the driest month (PDM) and of the coldest quarter (PCQ), and the soil variable, PC3. Hence, for the purposes of this study, we define ‘suitable habitat’ with respect to this combination of climate and soil variables. We note that the use of other variables may result in slightly different definitions and spatial extents of suitable habitat.

### Future habitat data

**Figure 1:**
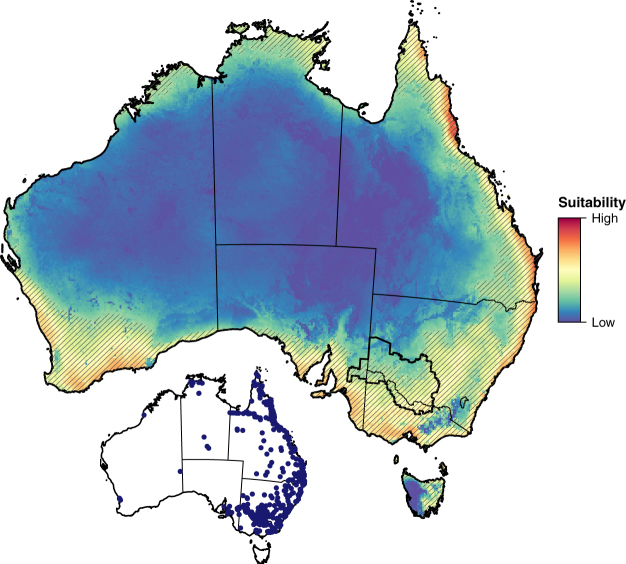
Current habitat suitability for Qfly modelled using Maxent (the hatched area represents regions with suitability values above the 10th percentile at training presence sites). The location of the former Fruit Fly Exclusion Zone (FFEZ) in south eastern Australia is shown as a polygon. The inset map shows the location of occurrence records of Queensland fruit fly (Qfly) from across Australia, based on specimens from natural history collections, literature and State Government-run trapping programs. Figure was created in R version 3.3.3^33^ (https://www.R-project.org/).

Given uncertainty in scenarios of future climate, impact assessments should incorporate data from a range of climate models that are effective in simulating historical climate over the area of interest. CSIRO compared the output of 40 global climate models (GCMs), and identified a subset of eight that they recommend for use in climate impact assessments^30^. These eight are representative of the range of results from all 40 models, for the Australian region, and are effective in reproducing historical conditions. Of the eight climate models, six had data at a resolution of 30 arc seconds, for the Representative Concentration Pathway 8.5 (RCP8.5)^31^. These models, and descriptions of changes they project for mean annual temperature (MAT) and annual precipitation (AP) for 2070, are as follows. (1) CanESM2 (The Second Generation of Canadian Earth System Model) projects an extremely hot, dry future, with warming >4 °C throughout central Australia, and >5.5 °C in parts of Western Australia. AP is projected to decline throughout central and Western Australia, and increase in north-east Queensland, with few changes in the south-east; (2) ACCESS1.0 (The Australian Community Climate and Earth System Simulator) projects a hot, dry future. Warming exceeds 2.5 °C across most of Australia, and >3.5 °C in central Australia. Drying is projected over most areas, including the horticultural zone in south-eastern Australia (see Fig. 1), although higher rainfall is likely in central Australia; (3) MIROC5 (Model for Interdisciplinary Research on Climate) projects moderate warming, not exceeding 3 °C, and slight changes in AP with declines in north-east Queensland and south-west Australia; (4) HadGEM2 (Hadley Centre Global Environmental Model Version 2) projects a hot future with warming typically >2.5 °C, and >3.5 °C in central regions. AP is projected to increase in central Australia, and decline elsewhere including the horticultural zone; (5) NorESM1 (The Norwegian Earth System Model-Part-1) projects moderate warming, with most of the continent exceeding 2 °C. Little change in AP is projected, particularly in the south-east, although there is drying in south-west WA; (6) GFDL-ESM2M (Global Coupled Climate Carbon Earth System Model Part-1) projects a hot, very dry future, with warming in central regions exceeding 3.5 °C. Drying is projected across most of the continent, with AP forecast to decline more than 20% in many areas.

We downloaded scenarios from these six models for the years 2030, 2050 and 2070 (http://www.climatechangeinaustralia.gov.au), and reprojected all data to a spatial resolution of 1 km × 1 km (Australian Albers Equal Area, EPSG: 3577) through bilinear interpolation, using the gdalwarp function provided by the R package gdalUtils^32^, in R version 3.3.3^33^.

### Species Distribution Model

Maxent (v3.3.3k^34^) is a machine learning algorithm frequently used to assess habitat suitability for species under current and future climate scenarios, because it accommodates presence-only data and has performed well in multi-model assessments^35^. Maxent produces a continuous probability surface, which can be interpreted as an index of habitat suitability given the predictor variables included in model calibration. Grid cells with a higher value are deemed as having greater suitability for the modelled species^34^. For detailed descriptions of Maxent, see^36,37^.

Maxent requires background data, to which it can compare the environmental characteristics of presence locations. Following Ihlow *et al*.^38^ we generated a mask layer consisting of a 200 km buffer surrounding Qfly occurrence records, from which Maxent randomly selected 10,000 background records. Our choice of background achieves a balance between fine-scale discrimination of suitable and unsuitable sites along environmental gradients, and generalisation of model predictions. In addition to comparing the predictive power of models calibrated with different sets of variables, we optimized Maxent by assessing the effect of different combinations of feature types and varying magnitudes of regularisation on model performance. We found that Maxent performed best when product, linear and quadratic features were used, with a regularization multiplier of 1, and used this configuration to calibrate our final model. We explored the contribution of environmental variables by a) assessing their permutation importance (i.e. the change in classifier accuracy when cell values for the respective variable are randomly permuted among presence and background cells) and b) with jackknife tests, which indicate the change in model fit or performance when sequentially withholding each predictor and refitting models, and when fitting univariate models^36^.

We used a ten-fold cross-validation to reduce model errors that may occur from the random splitting of data into test and training subsets. In this approach, occurrence data are split into ten subsets of approximately equal size (i.e. folds): the model is fitted using data from nine of the ten folds and tested using data from the remaining fold. This process is repeated until each fold has been used once for testing. The performance of each model was evaluated using the area under the receiver operating characteristic (ROC) curve (AUC), which describes the consistency with which a model ranks presence sites as more suitable than background sites. AUC ranges from 0 to 1^39^, where a value of 0.5 represents a model with discrimination ability no better than random, while a model with AUC > 0.75 is considered fair^40^.

### Current and future habitat suitability

To assess current and future habitat suitability, we projected the final Maxent model onto spatial data for each of the climate scenarios. Continuous suitability predictions were then converted into binary layers indicating suitable and unsuitable habitat. The selection of a threshold for this conversion depends on the goals of the study^41^ and the extent to which false negative and false positive errors are tolerated when identifying suitable habitat^39^. Following previous studies of pest species^23,42^, we selected the threshold corresponding to the 10th percentile of suitability at model-fitting presence localities. Data were then imported into ArcGIS (v 10.4, ESRI 2016). Binary layers were stacked to produce a consensus map, identifying agreement in the suitability of a grid cell across the six climate scenarios.

We obtained spatial data on the location of the former FFEZ from the Department of Primary Industries, Victoria. We also downloaded data on the primary horticultural regions of Australia, as mapped in the National Scale Land Use Version 5 (http://www.agriculture.gov.au/abares/aclump/land-use/data-download, 1 km resolution) developed by ACLUMP (Australian Collaborative Land Use and Management Program). ACLUMP contains spatial data on five types of horticultural regions (perennial; seasonal; irrigated perennial; irrigated seasonal; intensive horticultural), which span a total of 5,321 km^2^. We overlaid all Maxent projections for current and future time periods onto the FFEZ and horticultural regions, to assess the extent to which these areas are likely to contain suitable habitat for Qfly. Finally, for all scenarios we calculated overall range change, the proportion of current suitable habitat projected to become unsuitable (“loss”) and the proportion of future habitat projected to occur in previously unsuitable areas (“gain”).

### Data Availability

The datasets generated or analysed during the current study are available from the corresponding author on reasonable request. However, note that restrictions apply to data obtained from the Australian Plant Pest Database and Australian State Government Departments, which were used under license for the current study.

## Results

Across the ten cross-validation iterations, the average test AUC was 0.772 (SD 0.024). The most important variable was TminCM (36.5%), followed by MAT (33.3%) and PDM (14.9%). The remaining variables contributed <10% each to the model.

Our model suggested that approximately 23% of Australia is currently suitable for Qfly. Highly suitable habitat occurs along the east coast of Queensland and New South Wales, Victoria, southeastern South Australia, and southwestern Western Australia. Coastal zones of northern Western Australia, the Northern Territory and the eastern half of Tasmania have moderate suitability, while the arid/semi-arid zones of Western Australia and the Northern Territory are unsuitable (Fig. 1). Presently, ~64% of the FFEZ, spanning 120,589 km^2^ across the southeast of the zone, is suitable for Qfly (Fig. 1). Of the 5,321 km^2^ of land throughout Australia classified by ACLUMP as horticultural, ~97% is currently suitable for Qfly.

### Projections of climate change-driven shifts in habitat suitability

**Figure 2:**
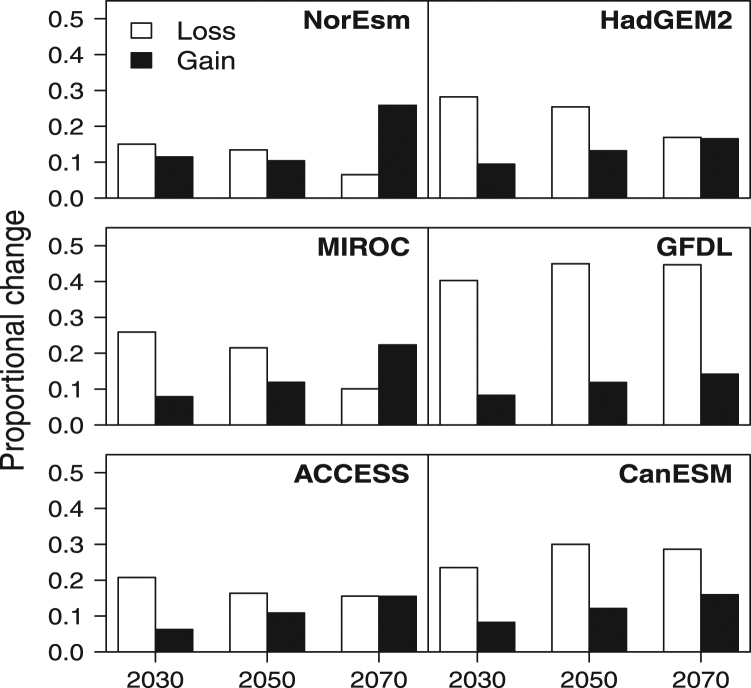
Projected changes in the area of suitable habitat for Queensland fruit fly, under six future climate scenarios, relative to the current period. Loss refers to the proportion of currently suitable habitat projected to become unsuitable in the future, while gain refers to the proportion of future suitable habitat that is in areas currently unsuitable.

The geographic extent of suitable habitat is projected to decline by 2030, by an average of 18.5% across the six scenarios (SD 10.0%), although as the century progresses, gains in new habitat may exceed losses under some scenarios (e.g. see NorESM and MIROC in Fig. 2). By 2070, the extent of suitable habitat is projected to be slightly larger, on average, than at present (mean = 1.2%, SD = 21.9%). However, there are considerable differences across climate scenarios. For example, under the hot/very dry scenario simulated by GFDL, total range size may decline ~35% by 2030, mostly due to contractions in the south and east, although limited gains in habitat may occur in northern Australia. Similarly, under the MIROC scenario, ~26% of current suitable habitat is projected to be lost by 2030, although by 2070, range expansions are projected to exceed losses. In contrast, few changes in overall range size are projected under NorESM (a moderate warming scenario with little precipitation change) by 2050, although by 2070, substantial westward range expansion is projected in eastern Australia.

### Agreement across climate scenarios

By 2030, ~25% of Australia (i.e. ~1,900,000 km^2^) is projected to be suitable for Qfly under at least one of the climate scenarios (Table 1). Due to subsequent gains in suitable habitat, this may increase to 31.7% (~2,400,000 km^2^) by 2070 (Table 1). Importantly, 12.7 to 14.2% (~979,000–1,088,000 km^2^) of Australia is likely to be suitable for Qfly by 2030 and 2070, under all six scenarios. This includes most of Victoria (with the exception of high altitude regions), much of eastern Tasmania, south-west Western Australia, eastern Queensland and the northern reaches of Australia.

**Table 1:** Area (km^2^) and percentage of Australia projected to be suitable for Queensland fruit fly under six future climate scenarios. That is, in the column ‘N. climate scenarios’, 0 refers to the area projected to be unsuitable across all six scenarios; 1 refers to the area projected to be suitable by any one of the six scenarios…6 refers to the area projected to be suitable under all six scenarios. The area of Australia is 7,687,258 km^2^.

N. climate scenarios	2030 km^2^	2030 (%)	2050 km^2^	2050 (%)	2070 km^2^	2070 (%)
0	5,767,276	75.02	5,755,445	74.87	5,252,171	68.32
1	321,497	4.18	205,527	2.67	429,639	5.59
2	125,806	1.64	147,398	1.92	192,653	2.50
3	111,207	1.44	145,441	1.89	168,931	2.19
4	140,535	1.82	222,494	2.89	303,480	3.94
5	241,687	3.14	215,993	2.81	251,669	3.27
6	979,250	12.74	994,960	12.94	1,088,715	14.16

**Figure 3:**
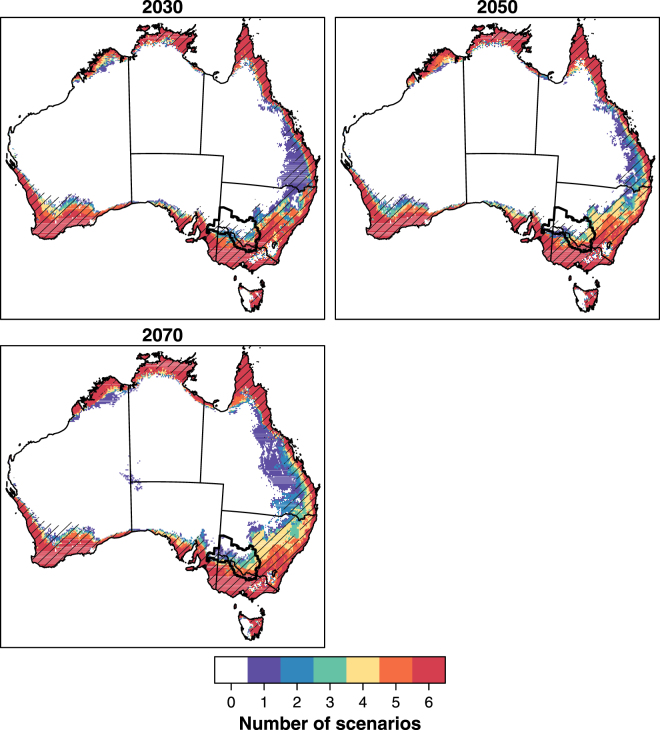
Agreement in the suitability of habitat for Queensland fruit fly across six climate scenarios for 2030, 2050 and 2070. Suitability was modelled with Maxent, and thresholded using the 10th percentile at training presence localities. Colours indicate the number of climate scenarios under which habitat is predicted to be suitable. The hatched area represents regions projected as suitable for the current period. Figure was created in R version 3.3.3^33^ (https://www.R-project.org/).

Within the former FFEZ, only the south-east region is projected as suitable across five or more scenarios for all time periods (Fig. 3). As the time horizon increases, however, the central and south-west regions of the exclusion zone become suitable under one to three scenarios (Fig. 3).

**Figure 4:**
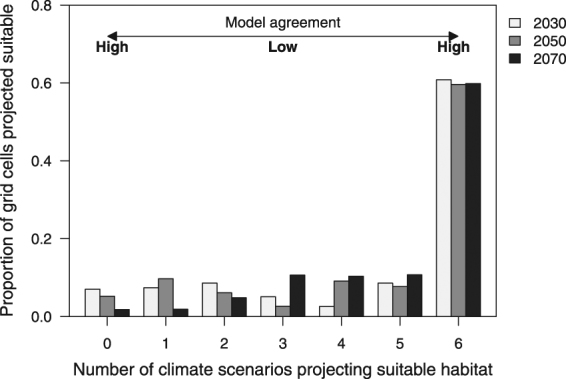
The proportion of grid cells that are suitable for Queensland fruit fly, and in which the primary land use is horticulture. Shown are six future climate scenarios for three time periods (2030, 2050, and 2070).

Approximately 60% of Australia’s current horticultural zones are projected to be suitable for Qfly across all climate scenarios for each time period (Fig. 4). An additional 11 to 21% is projected to be suitable under 4 or 5 of the climate scenarios, for 2030 and 2070, respectively.

## Discussion

Our study revealed substantial consensus across climate scenarios that south-eastern and south-western Australia will remain suitable for Qfly, until at least 2070. Similarly, eastern Tasmania, an island state currently free of Qfly, was classified as containing substantial areas of suitable habitat under both current climate and all future climate scenarios. Depending on which climate scenario eventuates, there is also the potential for large swaths of inland Queensland to become suitable by 2070. While the level of threat that Qfly may pose to the FFEZ varies with climate scenarios, the south-eastern regions of the FFEZ are likely to remain suitable across all scenarios, as are most of Australia’s current major horticultural regions. However, the northwest FFEZ is projected to be unsuitable until at least 2070.

Climate is considered ultimately responsible for determining the geographic distribution of Qfly^4^. According to our model for this species, the minimum temperature of the coldest month, mean annual temperature, and precipitation of the driest month are the variables with the greatest influence on suitability. This reflects known drivers of Qfly distribution. For instance, Muthuthantri *et al*.^43^ reported that many subtropical sites in Queensland are marginal for Qfly breeding and general activity in winter. Similarly, the southern extent of Qfly is limited by winter temperature^3^. In Melbourne, Qfly pupae do not generally survive winter months^2^. Hence, climate change driven increases in temperature of only 1–2 °C may substantially elevate the threat that Qfly poses to the horticultural industry in southern Australia^10^.

As climate changes, increases in temperature will affect the costs of Qfly management and losses incurred by growers. Sutherst *et al*.^10^ estimated that the cost to control Qfly within the FFEZ would increase by 24%, 33% and 83% for a 0.5 °C, 1.0 °C and 2.0 °C temperature increase, respectively, while for growers from Qfly endemic regions in Queensland control costs may increase by 42%, 47% and 82% under each of these scenarios. Among South Australian growers, expenditure on insect control and disinfestation was projected to increase by 34%, 63% and 114% for the three temperature scenarios, while the cost of management in Victoria may increase by 65%, 92% or 247%^10^. However, these figures were based on costs associated with spraying and disinfestation of pests. In 2011, the Australian Pesticides and Veterinary Medicines Authority substantially restricted the permitted usage patterns of insecticides used to control Qfly and other fruit pests, due to concerns about toxicity^44^. Pre-harvest use of organophosphate compounds, such as dimethoate and fenthion, was suspended or greatly reduced, while the post-harvest use of these chemicals was strictly restricted to a subset of fruits^45^. Consequently, other approaches to controlling Qfly outbreaks, such as sterile insect techniques, are now being explored. Given that a large extent of Australia’s current horticultural production regions will remain suitable for Qfly as climate changes, our results indicate a need for research and development into monitoring, control, and eradication tools. We do point out, however, that our analysis does not consider geographic shifts in horticultural zones that may occur due to climate change.

### Comparisons with other studies

In general, our results confirm those of Sutherst *et al*.^10^, who also predicted that Qfly will continue to pose a serious threat to the horticultural industry, particularly in southern Australia, as climate changes. As with CLIMEX^4^, Maxent projects northern regions of the Northern Territory, far north Queensland and eastern Queensland, as well as south-west Western Australia and southern South Australia, to be suitable for Qfly. Further, our model indicates that the southern region of the FFEZ is also suitable for Qfly, although Yonow and Sutherst’s^4^ model suggests this to be of marginal suitability. The primary difference between our projections and those of Yonow and Sutherst^4^ is that Maxent classifies much of Victoria and the eastern half of Tasmania as currently suitable whereas these areas were projected unsuitable by CLIMEX. However, there have been major outbreaks of Qfly in Victoria this century^46^ and it is clear that Qfly populations can now persist there, likely due to climate change-related warming and, potentially, increases in the level of cold tolerance of adults (Kalang *et al*. 2008, as reported in Holz *et al*.^9^).

More recent CLIMEX projections for Tasmania were undertaken by Holz *et al*.^9^. These results also contrasted with our model. Again, CLIMEX projected that permanent Qfly populations would not be able to establish in this state, although transient populations that may last several generations could occur if the fly was introduced into certain areas. The authors point out that because climate varies substantially across short distances in Tasmania, the spatial scale of modelling studies can influence results. Our analysis was conducted at a resolution of 1 km, an order of magnitude finer than Holz *et al*.^9^, who used grid sizes of 0.1 and 0.5 degrees.

Both Holz *et al*.^9^ and Sutherst *et al*.^10^ projected that climate suitability for Qfly in Tasmania and across southern Australia, respectively, will increase as climate change intensifies. Our models also indicate that these regions will be suitable until at least 2070, irrespective of the climate scenario. In particular, our results concur with Holz *et al*.’s projection of increased risks along the north and east coastlines of Tasmania^9^. We note, however, that Sutherst *et al*.’s^10^ models generally projected a far greater extent of mainland southern Australia to be suitable under current and future conditions than our model. In some respects, it is difficult to compare our results with those of Sutherst *et al*.^10^ who included irrigation when formulating their model. It is possible that our model’s projections of future habitat suitability for urban and horticultural areas may be altered should irrigation be incorporated. These two studies also utilised different baseline climate data sets and spatial resolutions (50 km versus 1 km).

Finally, Maxent and CLIMEX offer two very different approaches to modelling habitat suitability. As a correlative model, Maxent generates predictions based on statistical relationships between occurrence patterns and environmental data. In contrast, CLIMEX, a semi-mechanistic model, can be calibrated by setting parameter values that describe the species’ response to temperature and moisture either based on physiological data or inferred from the species’ known distribution^4^. A number of previous studies have compared the output of Maxent and CLIMEX for both invasive and non-invasive species. Most of these studies found the models to generate similar geographic extents of suitable habitat^22,47–49^. For example, Kumar *et al*.^48^ used both models to project the global distribution of the codling moth, *Cydia pomonella*, a major pest of pome and stone fruits. Both models’ projections reflected the current known distribution of the moth, although Maxent projected marginally suitable habitat to cover a greater geographic extent than CLIMEX projected. In contrast, Kumar *et al*.^50^ found that Maxent provided a more realistic model of the western cherry fruit fly, *Rhagoletis indifferens*, compared to CLIMEX, and suggested that differences in the suitability maps may have occurred due to different spatial resolutions (5 km, Maxent; 18 km, CLIMEX) and predictor variables (WorldClim, Maxent; CliMond, CLIMEX). We suggest that it would be very worthwhile to undertake a thorough comparison of projections for Qfly derived from both Maxent and CLIMEX.

## Model Limitations and Uncertainties

Errors and uncertainties in SDM output may occur for a variety of reasons, including limitations in occurrence data^51,52^, selection of background points^53,54^, spatial resolution, extent of the study area, and selection of predictor variables^55^. To minimise model errors, we (1) reduced the number of variables by assessing collinearity, (2) examined spatial autocorrelation and sampling bias before modelling, and (3) extracted background points from areas situated within 200 km of Qfly occurrences.

We converted continuous probability surfaces projected by Maxent to binary suitable/unsuitable maps, since this facilitates effective portrayal of model consensus. However, two types of errors occur in binary classification models. False negatives (or omission errors) occur when suitable habitat is classified as unsuitable, whereas false positives (or commission errors) are when unsuitable habitat is classified as suitable. Both can be costly when the output of models is used to support management decisions^56^. For invasive species, false negatives may translate into an underestimation of the geographic extent of suitable habitat, and hence, invasion risk. This may be particularly problematic if it results in poor decision-making^57^ such as allowing movement of goods^58^ or the failure to establish appropriate surveillance or containment measures^56^. In contrast, false positives may result in some locations being unnecessarily monitored^57^. The relative, application-specific importance of these errors is critical when selecting a threshold value at which to convert a continuous suitability map into a binary suitable/unsuitable map. In the context of Qfly, a precautionary approach would seem warranted: incorrectly labelling suitable habitat as unsuitable is particularly problematic, since the costs associated with uncontrolled incursions are likely to outweigh the costs of inadvertently monitoring an unsuitable site. Accordingly, we assumed that areas were ‘suitable’ if their predicted suitability was at least as high as the 10th percentile of suitability at presence localities. This ensures that the majority of conditions currently encountered by Qfly populations are considered suitable. However, it also accommodates some degree of positional error in occurrence data, and may exclude regions for which the occurrence records represent anomalies (e.g. populations that represent rare outbreaks or presences associated with transportation of goods, such as in central Australia and parts of the Northern Territory and western Queensland). Using a lower threshold increases the geographic extent of suitable habitat. For Qfly, this would result in suitability in Queensland and northern regions of the Northern Territory more closely aligning with Yonow and Sutherst’s^4^ model. However, it would also extend the distribution of suitable habitat in southern Australia, resulting in greater differences, compared to the projections made by Yonow and Sutherst^4^, for this region.

The selection of environmental predictor variables to be used in an SDM should be driven by the ecology and biology of the modelled species^59^. We used a set of general predictor variables related to soil and climate, yet other important environmental variables, such as host availability and dispersal, can influence species’ distributions. To some extent, we accounted for the influence of host availability by assessing changes in suitability within mapped horticultural regions. However, our incomplete knowledge of these aspects of species’ ecology means that including such variables remains a key challenge for modelling studies^60^. Hence, here we limit our focus to assessing the effects of climate change on climatic suitability.

## Conclusions

Our modelling projects that much of south-eastern and south-western Australia, eastern Queensland and Tasmania, as well the northern regions of Northern Territory, will likely be climatically suitable for Qfly throughout much of this century. As such, Qfly will remain a very real threat to Australia’s horticultural industry and backyard growers. For those markets that depend on area freedom, climate change may also translate into uncertainty about the security of market access^10^. Our projections provide guidance on the potential exposure of Australia’s horticultural industry to Qfly as a result of climate change, and highlight the need for long-term vigilance across southern Australia to prevent further range expansion of this species.
