# Author Correction: Potential impacts of climate change on habitat suitability for the Queensland fruit fly

**DOI:** 10.1038/s41598-018-23430-2

**Published:** 2018-04-12

**Authors:** Sabira Sultana, John B. Baumgartner, Bernard C. Dominiak, Jane E. Royer, Linda J. Beaumont

**Affiliations:** 1 Department of Biological Sciences, Macquarie University, North Ryde, New South Wales 2109 Australia; 2 New South Wales Department of Primary Industries, Locked Bag 21, Orange, New South Wales 2800 Australia; 3 Queensland Department of Agriculture and Fisheries, Biosecurity Queensland, Brisbane, Queensland 4001 Australia

*Correction to: Scientific Reports* 10.1038/s41598-017-13307-1; published online 12 October 2017

The original version of this Article contained errors in the Abstract.

“The Queensland fruit fly, *Bactrocera tryoni* Froggatt (Qfly), is the most economically damaging insect pest of Australia’s horticultural industry, and its management is a key priority for plant protection and biosecurity.”

now reads:

“The Queensland fruit fly, *Bactrocera tryoni* (Froggatt) (Qfly), is the most economically damaging insect pest of Australia’s horticultural industry, and its management is a key priority for plant protection and biosecurity.”

The original version of this Article also contained errors in the Introduction.

“The Queensland fruit fly (Qfly), *Bactrocera tryoni* Froggatt, is the most devastating pest of Australia’s $9 billion p.a. horticulture industry.”

now reads:

“The Queensland fruit fly (Qfly), *Bactrocera tryoni* (Froggatt), is the most devastating pest of Australia’s $9 billion p.a. horticulture industry.”

These errors have now been corrected in the PDF and HTML versions of the Article.

